# Supplementary material for: Streptococcal Arginine Deiminase Inhibits T Lymphocyte Differentiation In Vitro
Source: Microorganisms. 2023 Oct 19;11(10):2585. doi: 10.3390/microorganisms11102585 (PMC10608802; doi:10.3390/microorganisms11102585)
Supplement: Supplementary file 1 [file microorganisms-11-02585-s001.zip › microorganisms-2622924-supplementary.pdf]

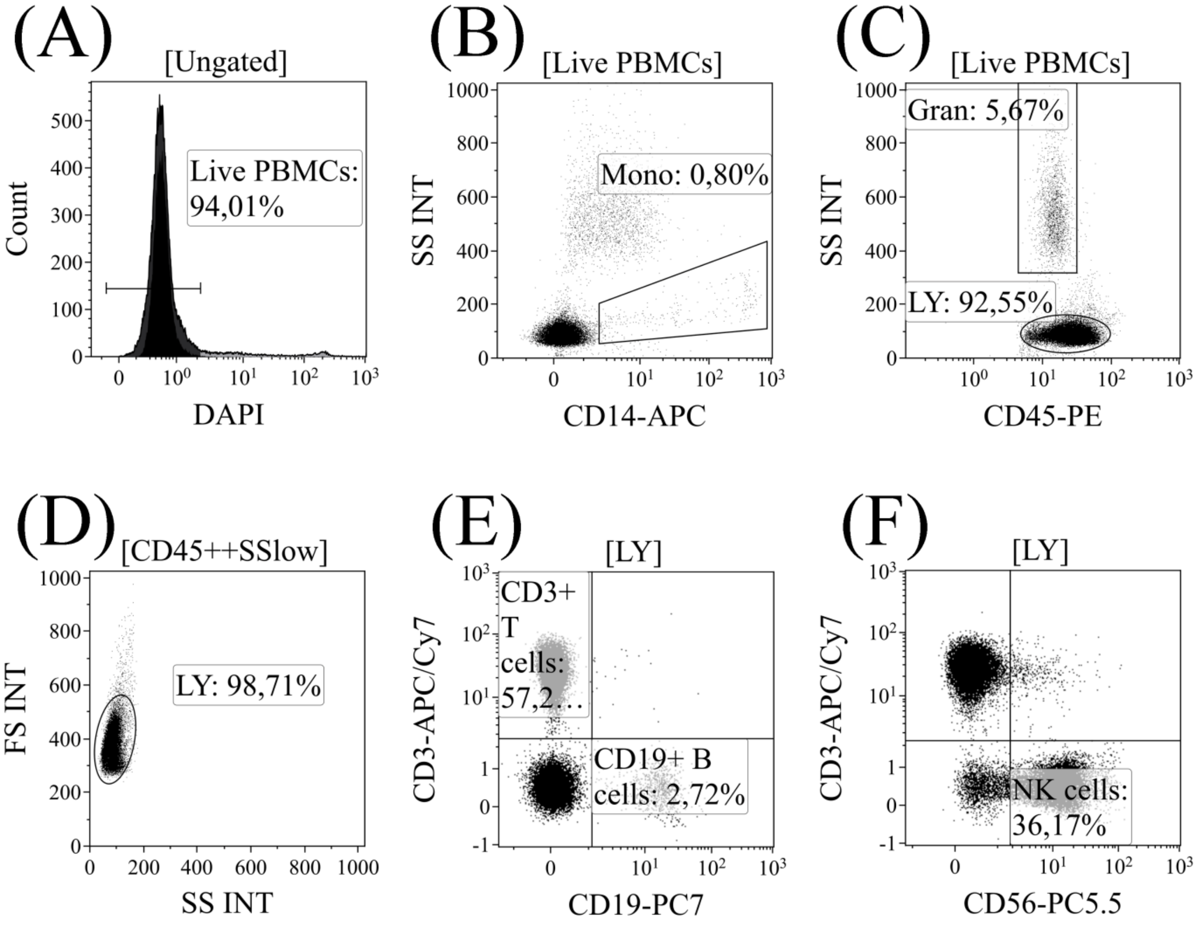

Figure S1. Flow cytometric analysis of PBMCs prior the *in vitro* experiments**.** Dot plot (A) live cell discrimination (DAPI solution (final concentration 1 µg/mL (DAPI was manufactured by BioLegend Inc, San Diego, CA, USA, cat# 422801) was used to discriminate alive (DAPI negative) and dead (DAPI positive) cells); dot plot (B) monocytes were detected as CD14-positive cells (APC-labeled mouse anti-human antibodies (cat# 301808, BioLegend Inc, San Diego, CA, USA); dot plot (C) total lymphocytes subset was identified as CD45brightSSlow, while total granulocytes was CD45brightSShigh (PE-labeled mouse anti-human CD45 antibody was manufactured by BioLegend Inc, San Diego, CA, USA, cat# 304008) (Note: the relative numbers of monocytes were 0.8% (0.5; 1.5) within alive PBMCs, the relative number of granulocytes were 3.8% (3.0; 5.0), while the relative numbers of lymphocytes were 94.5% (93.0; 95.0), n = 8, the group median and quartile ranges (Med (Q25; Q75) are shown). Dot plot (D) total lymphocyte subset identification based on side scatter and forward scatter. Next, we analyzed the frequencies of CD3+ T-cells, CD19+ B-cells and CD3-CD56+ NK-cells on dot plots (E) and (F). We used APC/Cy7-labeled mouse anti-human CD3 antibodies (cat# 317342), PE/Cy7-labeled mouse anti-human CD19 antibodies (cat# 302216) and PerCP/Cyanine5.5-labeled mouse anti-human CD56 antibodies (cat# 362506), all antibodies were manufactured by BioLegend Inc, San Diego, CA, USA and used according manufactories recommendations. **Note**: within the total lymphocytes subset the frequency of CD3+ was 63.0% (58.0; 70.0), the frequency of CD19+ B-cells was 11.0% (10.0; 15.0), while the frequency of CD3-CD56+ NK-cells was 20.5% (14.0; 30.0) (n = 8, the group median and quartile ranges (Med (Q25; Q75) are shown). Sample acquisition was performed using a 3/10 Navios flow cytometer (Beckman Coulter, Indianapolis, IN, USA). The flow cytometry data were analyzed using Kaluza software v2.3 (Beckman Coulter, Indianapolis, IN, USA).

Table S1. Time-dependent arginine concentration in cell culture supernatants after exposure to parental and mutant strain-derived SDSC.

Arginine supplement	Incubation duration. (hours)	L-arginine concentration (μM, Med [Q25;Q75], n=8) in the medium after PBMCs incubation in the presence			L-arginine concentration (μM, Med [Q25;Q75] n=8) in the medium after PBMCs stimulation in the presence		
		**Culture medium (control)**	**M49-16**	**M49-16 delArcA**	**Culture medium (control)**	**M49-16**	**M49-16 delArcA**
0 μM	24	805.3 [737.2;831.0]	132.3 [52.9;139.0] ΔΔ	765.6 [684.3;790.0] ††	875.2 [790.2;888.0]	138.0 [3.8;189.0] &&	865.8 [722.1;994.0] ••
	48	609.1 [537.2;664.9]	175.3 [136.3;208.7] Δ	657.2 [643.0;733.9] †	590.6 [547.7;683.0]	104.9 [63.9;188.7] &&	520.2 [495.9; 597.3] ••
	72	814.5 [741.5;879.5]	244.0 [215.5;320.5] ΔΔ	705.0 [642.5;748.0] ††	766.5 [715.5;877.5]	337.0 [294.5;364.5] &&	724.5 [618.5;828.5] ••
	96	1015.2 [978.5;1057.0]	243.9 [236;325.0] ΔΔ	852.9 [580.7;876] †	905.3 [763.1;1076.0]	261.8 [215.9;320.0] &&	929.0 [902.4;1003.0] ••
2 μM	24	1638.9 [1595.5;1716.0] ΔΔ	187.1 [124.8;820.0]	1833.6 [1795.8;1897.0] §§	2020.8 [1890.3;2071.0] &&	179.6 [136.1;283.0]	1763.7 [1640.8;1871.0] □□
	48	1278.2 [1121.8;1425.0] ΔΔ	124.9 [116.3;135.3]	1531.5 [1494.4;1662.0] §	1399.6 [1303.9;2024.9] &&	136.3 [112.5;152.5]	1188.7 [969.6;1762.0] □□
	72	1638.5 ΔΔ [1575;1699.5]	291.0 [170.5;395.5]	1518.5 [1220.5;1671.0] §§§	1725.0 [1519.5;1782.5] &&	310.5 [266.5;359.5]	1808.5 [1578.5;1890.5] □□
	96	2126.8 [2074.4;2195.0] ΔΔ	631.7 [570.6;715.0] †	2141.0 [1679.0;2266.0] §§	1385.0 [1107.8;2106.0] &	452.1 [312.1;777.0]	1652.2 [1498.5;1959.0] □□

Comments. Digitals 1, 2, 3 denote significance value at $p<0.05$, $p<0.01$, $p<0.001$, respectively.
Δ significant difference compared to control, without stimulation, in arginine-free settings;
† significant difference compared to M49-16, without stimulation, in arginine-free settings;
§ significant difference compared to M49-16delArcA, without stimulation, in arginine-free settings;
& significant difference compared to control with stimulation in arginine-free settings;
• significant difference compared to M49-16, with stimulation, in arginine-free settings;
□ significant difference compared to M49-16delArcA with stimulation in arginine-free settings.

Table S2. Time-dependent PBMC activation after exposure to parental and mutant strain-derived SDSC.

Arginine supplement	Incubation duration. (hours)	Activation PBMCs (%, Med [Q25;Q75], n=9) after incubation in the presence			Activation PBMC (%, Med [Q25;Q75], n=9) after stimulation in the presence		
		Culture medium (control)	M49-16	M49-16 delArcA	Culture medium (control)	M49-16	M49-16 delArcA
0 μM	24	100.0 [100.0;100.0]	99.7 [95.6;110.6]	109.6 [100.2;115.7] Δ	114.5 [108.0;115.6] ΔΔΔ	110.1 [95.1;111.0]	116.2 [110.7;122.5]
	48	100.0 [100.0;100.0]	105.3 [100.6;106.5]	114.2 [110.0;121.7] ΔΔΔ †	195.0 [160.2;212.6] ΔΔΔ	108.1 [101.5;128.2] &&&	165.5 [160.0;183.5] § ●●●
	72	100.0 [100.0;100.0]	102.7 [92.7;106.3]	143.2 [139.7;147.6] ΔΔΔ †††	229.1 [208;346.2] ΔΔΔ	101.1 [100.3;124.2] &&&	222.0 [216.2;254.3] §§§ ●●●
	96	100.0 [100.0;100.0]	124.1 [109.0;130.7] ΔΔΔ	245.5 [192.6;259.0] ΔΔΔ †††	308.6 [252.7;324.2] ΔΔΔ	114.3 [106.7;122.9] &&&	310.0 [262.4;319.5] § ●●●
2 μM	24	105.6 [100.3;110.6]	97.8 [90.5;106.0]	103.3 [95.3;105.8]	115.3 [112.5;122.5]	109.0 [106.9;112.1]	112.6 [111.5;115.3]
	48	101.8 [99.6;111.0] Δ	108.9 [103.5;116.1]	112.8 [108.1;128.9]	171.6 [160.0;195.6]	136.4 [122.0;146.9] ●	163.0 [156.7;172.5]
	72	96.9 [93.3;100.5] Δ	112.8 [104.1;117.6] †	144.7 [133.9;151.6]	248.4 [234.2;306.4]	129.0 [113.5;141.6] ●	238.7 [226.4;287.9] □
	96	105.4 [95.5;110.4]	133.3 [115.4;135.1] ††	240.1 [202.2;253.8]	413.0 [276.1;448.7]	145.8 [129.3;153.1] ●●	370.3 [276.5;382.6] □

Comments. Digitals 1, 2, 3 denote significance value at p<0.05, p<0.01, p<0.001, respectively.
Δ significant difference compared to control, without stimulation, in arginine-free settings;
† significant difference compared to M49-16, without stimulation, in arginine-free settings;
§ significant difference compared to M49-16delArcA, without stimulation, in arginine-free settings;
& significant difference compared to control with stimulation in arginine-free settings;
• significant difference compared to M49-16, with stimulation, in arginine-free settings;
□ significant difference compared to M49-16delArcA with stimulation in arginine-free settings.

Table S3. IL-2 concentration in cell culture supernatants after exposure to parental and mutant strain-derived SDSC.

Arginine supplement	IL-2 concentration (pg/ml, Med [Q25;Q75], n=7) after 96 hours PBMCs incubation in the presence			IL-2 concentration (pg/ml, Med [Q25;Q75], n=7) after 96 hours PBMC stimulation and incubation in the presence		
	Culture medium (control)	M49-16	M49-16 delArcA	Culture medium (control)	M49-16	M49-16 delArcA
0 μM	1.53 [1.53; 1.53]	0.90 [0.68; 1.32]	1.53 [0.90; 3.87]	22.3 [14.3; 27;1] Δ	8.98 [6.00; 13.45] &	17.70 [6.64; 23.66] §
2 μM	1.32 [0.89; 2.60]	1.53 [1.11; 2.17]	2.60 [1.11; 2.81]	16.21 [8.34 [21.53]	13.87 [6.85; 18.77]	16.85 [11.75; 17.70]

Comments. Digitals 1, 2, 3 denote significance value at $p<0.05$, $p<0.01$, $p<0.001$, respectively.
Δ significant difference compared to control, without stimulation, in arginine-free settings;
§ significant difference compared to M49-16delArcA, without stimulation, in arginine-free settings;
& significant difference compared to control with stimulation in arginine-free settings.

Table S4. Effects of parental and mutant strain-derived SDSC on CD8+ T cell differentiation (48-hour incubation).

Arginine supplement	CD8+ Subsets	Proportion of cells (%, Med [Q25;Q75], n=13) after 48 hours PBMCs incubation in the presence			Proportion of cells (%, Med [Q25;Q75], n=13) after 48 hours PBMC stimulation in the presence		
		Culture medium (control)	M49-16	M49-16 delArcA	Culture medium (control)	M49-16	M49-16 delArcA
0 µM	Naïve	68.2 [64.6;70.9]	73.1 [63.6;76.2]	74.9 [68.3;81.0]	56.0 [41.0;77.0]	63.5 [56.2;71.6]	51.1 [47.2;71.8] §
	CM	7.8 [6.0;13.1]	5.5 [4.1;11.2]	5.3 [4.2;9.7]	7.3 [3.7;8.9]	4.2 [3.6;8.2]	7.1 [5.1;10.0]
	EM	12.5 [10.3;16.8]	9.7 [8.9;14.0]	7.2 [6.9;11.8] Δ	8.8 [6.3;15.8]	8.8 [7.5;15.7]	10.2 [8.6;16.3]
	TEMRA	9.6 [9.1;15.8]	11.6 [8.7;15.9]	8.9 [8.3;15.4]	19.6 [11.3;26.3] Δ	18.4 [16.4;24.4] †	24.5 [15.9;30.4] §§
2 µM	Naïve	67.4 [59.2;69.2]	72.9 [63.4;76.4]	71.4 [66.2;78.5]	51.1 [46.5;68.8]	50.8 [45.9;54.2]	52.2 [41.9;69.5]
	CM	7.6 [6.3;12.6]	5.4 [4.7;10.2]	5.5 [4.6;10.7]	6.8 [4.4;9.9]	6.0 [3.9;9.3]	6.3 [4.6;10.4]
	EM	13.6 [11.4;17.2]	10.5 [8.0;15.0]	8.4 [7.5;13.4]	10.0 [8.0;18.4]	12.3 [10.3;20.8]	11.7 [9.1;15.3]
	TEMRA	12.1 [9.5;16.1]	12.4 [8.2;14.1]	11.0 [8.8;15.2]	20.4 [14.1;32.1]	25.0 [19.6;29.4]	23.5 [14.3;31]

Comments. Digitals 1, 2, 3 denote significance value at $p<0.05$, $p<0.01$, $p<0.001$, respectively.
Δ significant difference compared to control, without stimulation, in arginine-free settings;
† significant difference compared to M49-16, without stimulation, in arginine-free settings;
§ significant difference compared to M49-16delArcA, without stimulation, in arginine-free settings;
& significant difference compared to control with stimulation in arginine-free settings;
• significant difference compared to M49-16, with stimulation, in arginine-free settings.

Table S5. Effects of parental and mutant strain-derived SDSC on CD8+ T cell differentiation (96-hour incubation).

Arginine supplement	CD8+ Subsets	Proportion of cells (%, Med [Q25; Q75], n=13) after 96-hour PBMC incubation in the presence			Proportion of cells (%, Med [Q25; Q75], n=13) after 96-hour PBMC stimulation in the presence		
		Culture medium (control)	M49-16	M49-16 delArcA	Culture medium (control)	M49-16	M49-16 delArcA
0 μM	Naïve	55.7 [41.7;66.7]	59.7 [51.2;69.3]	57.7 [45.1;68.8]	30.3 [22.5;51.5] ΔΔ	49.2 [42.3;60.6] † &	30.4 [22.4;51.9] §§ ●
	CM	11.2 [8.7;18.5]	8.6 [7.1;12.8] Δ	9.9 [7.1;14.0]	34.5 [28.3;46.2] ΔΔΔ	9.3 [5.8;14.0] &&&	40.9 [30;48.2] §§§ ●●●
	EM	18.5 [11.5;20.4]	16.4 [8.6;21.2]	13.5 [9.7;18.4]	20.3 [10.1;27.8]	17.9 [11.8;22.8]	15.8 [12.3;24.0]
	TEMRA	9.8 [6.1;16.7]	12.3 [9.2;17.1]	14.4 [7.9;18.6]	5.1 [3.8;8.7] Δ	21.2 [14.7;30.3] †† &&&	4.7 [3.1;7.6] §§§ ●●●
2 μM	Naïve	59.2 [49.4;68.3]	60.9 [49.9;70.7]	57.0 [44.5;68.5]	30.9 [16.2;46.8]	37.5 [16.7;49.1] ●	34.2 [12.0;52.1]
	CM	10.2 [9.0;14.7]	10.2 [5.9;14.6]	10.8 [8.0;16.2]	44.6 [32.4;51.5]	28.3 [10.5;39.2] ●●	42.4 [29.1;60.7]
	EM	15.4 [12.5;22.4]	14.6 [10.6;19.5]	14.7 [12.0;18.2]	15.1 [9.9;24.4]	18.9 [15.5;27.8]	13.0 [8.5.0;24.7]
	TEMRA	9.8 [7.1;16.9]	11.0 [8.4;17.9]	11.4 [8.8;14.1]	3.8 [2.7;6.2]	12.1 [4.2;22.7] ●	3.4 [2.6;7.3]

Comments. Digitals 1, 2, 3 denote significance value at p<0.05, p<0.01, p<0.001, respectively.
Δ significant difference compared to control, without stimulation, in arginine-free settings;
† significant difference compared to M49-16, without stimulation, in arginine-free settings;
§ significant difference compared to M49-16delArcA, without stimulation, in arginine-free settings;
& significant difference compared to control with stimulation in arginine-free settings;
• significant difference compared to M49-16, with stimulation, in arginine-free settings.

Table S6. Effects of parental and mutant strain-derived SDSC on CD4+ T cell differentiation (48-hour incubation).

**Arginine supplement**	**CD4+ Subsets**	**Proportion of cells (%, Med [Q25;Q75], n=13) after 48hours PBMCs incubation in the presence**			**Proportion of cells (%, Med [Q25;Q75], n=13) after 48 hours PBMC stimulation in the presence**		
		**Culture medium (control)**	**M49-16**	**M49-16 delArcA**	**Culture medium (control)**	**M49-16**	**M49-16 delArcA**
0 μM	Naïve	56.1 [47.3;58.2]	58.4 [54.1;65.2]	66.1 [59.5;70.4] ΔΔ	53.9 [47.2;66.4]	68.4 [65.6;77.1] † &&&	55.2 [47.5;63.7] •••
	CM	33.3 [27.4;46.7]	27.7 [24.9;41.5]	25.6 [23.0;37.5]	25.9 [18.8;41.1]	23.5 [19.9;32.1] †	26.3 [22;43.7]
	EM	11.0 [8.9;11.5]	9.0 [6.4;10.3]	6.5 [4.6;9.6] ΔΔ	6.8 [5.3;14.7]	4.1 [2.8;7.7] &&	10.0 [7;14.4] ••
	TEMRA	0.7 [0.5;1.3]	0.8 [0.4;1.4]	0.5 [0.3;0.6]	2.0 [1.5;2.5] ΔΔΔ	1.8 [1.4;2.3] †	2.6 [1.8;6.3] §§§
2 μM	Naïve	55.0 [49.1;56.7]	60.7 [57.3;66.8]	61.7 [54.8;68.0]	55.7 [42.3;59.5]	52.0 [42.2;58] •	53.0 [37.1;60.0]
	CM	32.5 [29.6;44.0]	28.8 [25;38.7]	27.7 [25.9;41.3]	31.2 [21.2;45.3]	27.1 [22.6;40.8]	28.5 [21.8;44.8]
	EM	10.7 [9.1;11.9]	7.4 [5.6;9.8]	6.3 [4.4;10.1]	9.6 [6.2;14.1]	11.5 [7.9;15.6] •••	8.5 [6.7;15.0]
	TEMRA	0.6 [0.5;1.2]	0.7 [0.4;1.1]	0.5 [0.2;0.6]	2.5 [1.9;4.9]	4.6 [2.2;7.3] •	3.0 [1.5;6.3]

Comments. Digitals 1, 2, 3 denote significance value at $p<0.05$, $p<0.01$, $p<0.001$, respectively.
Δ significant difference compared to control, without stimulation, in arginine-free settings;
† significant difference compared to M49-16, without stimulation, in arginine-free settings;
§ significant difference compared to M49-16delArcA, without stimulation, in arginine-free settings;
& significant difference compared to control with stimulation in arginine-free settings;
• significant difference compared to M49-16, with stimulation, in arginine-free settings.

Table S7. Effects of parental and mutant strain-derived SDSC on CD4+ T cell differentiation (96-hour incubation).

Arginine supplement	CD4+ Subsets	Proportion of cells (%, Med [Q25;Q75], n=13) after 96-hour PBMCs incubation in the presence			Proportion of cells (%, Med [Q25;Q75], n=13) after 96-hour PBMC stimulation in the presence		
		**Culture medium (control)**	**M49-16**	**M49-16 delArcA**	**Culture medium (control)**	**M49-16**	**M49-16 delArcA**
0 μM	Naïve	43.1 [37.1;49.3]	40.7 [36.8;54.4]	44.3 [39.5;50.9]	16.0 [8.7;20.4] ΔΔΔ	39.8 [33.3;50.6] &&&	16.0 [9.2;18.6] §§§ ●●●
	CM	45.3 [36.5;48.9]	43.5 [32.2;47.9]	43.8 [36.4;52.3]	66.5 [54.2;71.2] ΔΔΔ	44.4 [32.9;46.9] &&&	68.9 [60.9;75.1] §§§ ●●●
	EM	12.3 [9.3;15.2]	11.9 [9.1;14.1]	10.5 [8.2;12.7]	14.1 [9.2;24.8]	12.3 [8.9;17.2]	11.3 [9.1;21]
	TEMRA	0.8 [0.4;1.0]	1.4 [0.9;1.9]	0.8 [0.6;1.7]	1.2 [0.7;2.0] Δ	3.4 [2.2;5.9] ††† &&&	1.1 [0.6;1.9] ●●●
2 μM	Naïve	43.5 [39.0;52.0]	44.0 [38.3;52.4]	42.2 [38.2;49]	12.0 [7.8;19.4]	22.7 [13.6;32.1] ●●●	10.3 [7.6;16.2]
	CM	42.2 [36.4;50.7]	42.7 [37.2;47.9]	45.1 [37.5;49.2]	72.5 [62.2;81.7]	56.8 [48.1;70.2] ●●●	73.3 [67.2;79.6]
	EM	11.3 [8;14.7]	11.7 [8.0;13.2]	9.9 [8.5;13.1]	10.0 [6.5;18.1]	12.9 [10.0;18.5]	10.6 [6.1;18.2]
	TEMRA	0.6 [0.4;1.0]	1.2 [0.8;2.3] Δ	0.8 [0.5;1.8]	0.6 [0.3;1.0] &	2.0 [0.9;3.5] ●●●	0.6 [0.5;0.9]

Comments. Digitals 1, 2, 3 denote significance value at $p<0.05$, $p<0.01$, $p<0.001$, respectively.
Δ significant difference compared to control, without stimulation, in arginine-free settings;
§ significant difference compared to M49-16delArcA, without stimulation, in arginine-free settings;
& significant difference compared to control with stimulation in arginine-free settings;
• significant difference compared to M49-16, with stimulation, in arginine-free settings.

Table S8. Effects of parental and mutant strain-derived SDSC on CD8+ T cell proliferation (96-hour incubation).

Arginine supplement	CD8+ Subsets	Division index (%, Med [Q25; Q75], n=28) after PBMCs incubation in the presence			Division index (%, Med [Q25; Q75], n=28) after PBMC stimulation and incubation in the presence		
		**Culture medium (control)**	**M49-16**	**M49-16 delArcA**	**Culture medium (control)**	**M49-16**	**M49-16 delArcA**
0 μM	Naïve	2.4 [2.1; 4.0]	4.0 [2.8; 4.2]	2.2 [2.2; 3.6]	9.1 [4.3; 11.5] Δ	2.1 [2.1; 3.6] † &&	5.2 [3.4; 7.3] § ●●
	CM	2.9 [2.0; 4.0]	2.3 [2.1; 2.5]	2.8 [2.0; 5.5]	12.1 [7.7; 16.3] Δ	1.0 [0.0; 2.4] &&	15.3 [8.6; 18.9] §§ ●●
	EM	2.2 [2.0; 7.5]	2.0 [2.0; 2.1]	2.3 [2.0; 5.5]	9.7 [8.3; 15.5] Δ	2.0 [2.0; 2.17] &&	12.2 [9.1; 18.6] § ●●●
	TEMRA	2.0 [0.5; 3.5]	2.0 [0.0; 2.1]	2.0 [0.5; 2.7]	8.5 [4.4; 13.4] Δ	1.0 [0.0; 2.0] &&&	5.9 [5.1; 7.2] §§ ●●
2 μM	Naïve	3.0 [2.1; 4.1]	3.9 [2.3; 4.1]	2.3 [2.1; 4.0]	3.8 [2.9; 6.9] &	3.0 [2.1; 4.4]	4.4 [3.6; 6.3]
	CM	2.1 [2.0; 2.9]	2.8 [2.0; 4.4]	2.5 [2.1; 9.8]	12.5 [7.2; 18.0]	3.8 [2.4; 11.1] ●●	9.8 [5.38; 11.1]
	EM	2.0 [2.0; 2.1]	2.0 [2.0; 4.3]	2.3 [2.0; 4.2]	11.8 [7.6; 13.2]	4.2 [3.2; 10.1] ●●●	11.4 [7.5; 15.0]
	TEMRA	2.0 [0.0; 2.1]	2.0 [0.5; 2.2]	2.0 [2.0; 2.3]	4.65 [3.0; 6.0] &	3.3 [2.0; 6.5] ●	3.8 [2.7; 5.3] □

Comments. Digitals 1, 2, 3 denote significance value at $p<0.05$, $p<0.01$, $p<0.001$, respectively.
Δ significant difference compared to control, without stimulation, in arginine-free settings;
§ significant difference compared to M49-16delArcA, without stimulation, in arginine-free settings;
& significant difference compared to control with stimulation in arginine-free settings;
• significant difference compared to M49-16, with stimulation, in arginine-free settings;
□ significant difference compared to M49-16delArcA with stimulation in arginine-free settings.

Table S9. Effects of parental and mutant strain-derived SDSC on CD4+ T cell proliferation (96 hour incubation).

Arginine supplement	CD4+ Subsets	Division index (%, Med [Q25;Q75], n=28) after PBMCs incubation in the presence			Division index (%, Med [Q25;Q75], n=28) after PBMC stimulation and incubation in the presence		
		Culture medium (control)	M49-16	M49-16 delArcA	Culture medium (control)	M49-16	M49-16 delArcA
0 μM	Naïve	4.1 [3.8; 4.4]	3.7 [3.6; 4.0]	4.1 [3.7; 4.6]	4.4 [3.6; 6.5]	3.9 [2.5; 4.0]	3.7 [2.7; 6.1]
	CM	3.9 [2.2; 4.1]	3.9 [2.1; 4.0]	3.1 [2.1; 4.2]	8.1 [7.1; 9.7] ΔΔΔ	2.1 [2.0; 4.0] &&&	7.3 [6.8; 9.3] §§§ ●●●
	EM	2.2 [2.0; 4.4	2.0 [2.0; 2.9]	3.1 [2.0; 6.2]	8.9 [8.3; 10.78] ΔΔΔ	2.1 [2.0; 2.3] &&&	9.7 [8.2; 14.9] §§ ●●●
	TEMRA	2.0 [0.5; 4.9]	2.3 [2.0; 4.0]	2.5 [2.1; 3.9]	4.7 [3.9; 7.9] Δ	2.1 [2.0; 2.1] &&&	4.3 [3.7; 7.1] § ●●●
2 μM	Naïve	4.0 [3.7; 4.2]	4.0 [3.9; 4.5]	4.0 [3.7; 4.3]	4.2 [2.8; 5.0]	3.4 [2.7; 4.2]	3.6 [2.9; 5.0]
	CM	3.9 [2.4; 4.2]	2.9 [2.1; 3.9]	2.9 [2.1; 4.0]	8.8 [5.7; 11.0]	5.3 [2.4; 9.0] ●	11.1 [8.2;13.2] □
	EM	2.2 [2.0; 2.5]	2.2 [2.0; 3.0]	3.3 [2.1; 4.3]	7.1 [5.9; 10.0]	6.5 [4.2; 9.5] ●●●	7.8 [5.4; 8.8] □
	TEMRA	2.2 [2.0; 2.8]	2.2 [2. 1; 2.6]	2.7 [2.2; 3.7]	3.8 [3.2; 6.15]	3.3 [2.7; 3.6] ●●●	3.6 [2.9; 5.0]

Comments. Digitals 1, 2, 3 denote significance value at $p<0.05$, $p<0.01$, $p<0.001$, respectively.
Δ significant difference compared to control, without stimulation, in arginine-free settings;
§ significant difference compared to M49-16delArcA, without stimulation, in arginine-free settings;
& significant difference compared to control with stimulation in arginine-free settings;
• significant difference compared to M49-16, with stimulation, in arginine-free settings;
□ significant difference compared to M49-16delArcA with stimulation in arginine-free settings.

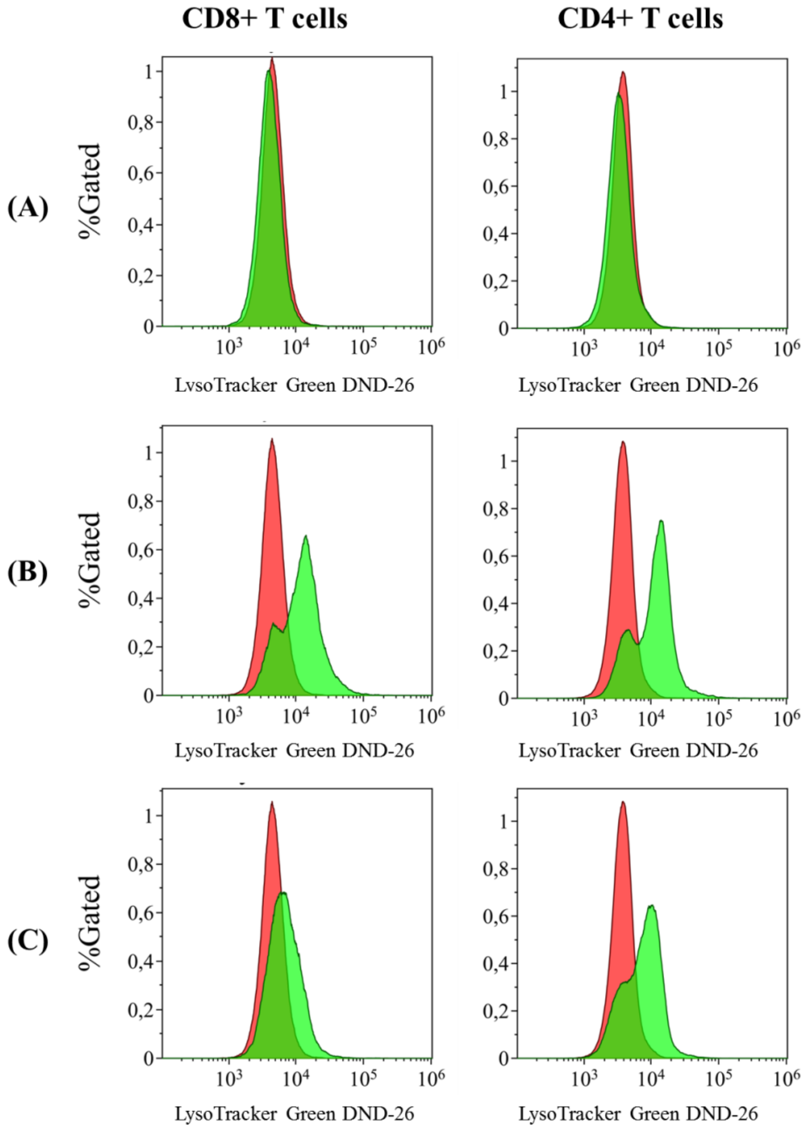

Figure S2. Effects of chloroquine (CQ) on anti-CD2/CD3/CD28-induced T cell autophagy**.** Representative histograms reflecting changes in the autophagy level (A) Ctrl (Red), 10 μM CQ treatment (Green); (B) Ctrl (Red), anti-CD2/CD3/CD28 treatment (Green) (C) Ctrl (Red), anti-CD2/CD3/CD28 + CQ 10 μM treatment (Green). PBMCs were activated with anti-CD2/CD3/CD28 loaded beads in the presence or absence of 10 μM CQ for the last 20 h. After 48 hours cells were stained with antibody cocktail (see 2.6), samples were added with dye Lysotracker Green DND-26. To exclude necrotic cells from the analysis, the samples were stained with DAPI. Samples were analyzed by flow cytometry. The results were expressed as mean of fluorescence intensity (MFI).

Table S10. Effects of parental and mutant strain-derived SDSC on CD8+ T cell autophagy (48 hour incubation).

Arginine supplement	CD8+ Subsets	Intensity of autophagy (MFI, Med [Q25;Q75], n=13) after 48 hours PBMCs incubation in the presence			Intensity of autophagy (MFI, Med [Q25;Q75], n=13) after 96 hours PBMC stimulation in the presence		
		**Culture medium (control)**	**M49-16**	**M49-16 delArcA**	**Culture medium (control)**	**M49-16**	**M49-16 delArcA**
0 µM	Naïve	3.4 [3.1;3.8]	3.4 [3.2;4.0]	3.8 [3.5;4.2]	5.8 [4.3;6.7] ΔΔ	5.1 [4.7;5.4] ††	6.1 [4.7;9.3] §§
	CM	3.9 [2.6;4.3]	3.6 [3.4;4.0]	3.9 [3.2;4.3]	6.2 [5.2;7.2] ΔΔ	4.9 [4.3;5.3] &	6.9 [4.7;9.5] §
	EM	3.6 [2.8;4.1]	3.4 [3.1;4.4]	3.9 [3.2;4.4]	6.0 [5.2;7.4] ΔΔ	4.1 [3.7;5.4] &	6.2 [4.9;8.1] §§ &
	TEMRA	4.0 [3.2;5.4]	3.8 [3.7;5.2]	4.3 [3.8;5.7]	6.1 [5.9;7.0] Δ	5.3 [4.7;6.0]	6.3 [5.3;7.2] §
2 µM	Naïve	3.9 [3.6;4.1]	3.9 [3.2;4.0]	3.7 [3.5;4.3]	6.3 [4.9;8.4]	6.0 [4.8;6.9]	6.3 [4.8;8.6]
	CM	3.4 [3.1;4.2]	4.0 [3.1;4.5]	3.5 [3.2;4.2]	6.8 [5.0;9.3]	6.1 [5.4;8.0] •	6.8 [5.0;9.2]
	EM	3.7 [3.3;3.9]	4.0 [3.3;4.3]	3.5 [3.1;3.9]	6.4 [6.0;8.2]	6.7 [6.3;8.7] ••	6.7 [5.4;8.1]
	TEMRA	4.3 [3.6;5.0]	4.4 [4;5.3.0]	4.1 [3.7;5.2]	6.5 [5.8;7.4]	7.4 [5.7;8.0]	7.1 [5.4;7.3]

Comments. Digitals 1, 2, 3 denote significance value at $p<0.05$, $p<0.01$, $p<0.001$, respectively.
Δ significant difference compared to control, without stimulation, in arginine-free settings;
† significant difference compared to M49-16, without stimulation, in arginine-free settings;
§ significant difference compared to M49-16delArcA, without stimulation, in arginine-free settings;
& significant difference compared to control with stimulation in arginine-free settings;
• significant difference compared to M49-16, with stimulation, in arginine-free settings.

Table S11. Effects of parental and mutant strain-derived SDSC on CD8+ T cell autophagy (96-hour incubation).

Arginine supplement	CD8+ Subsets	Intensity of autophagy (MFI, Med [Q25;Q75], n=13) after 96hours PBMCs incubation in the presence			Intensity of autophagy (MFI, Med [Q25;Q75], n=13) after 96-hour PBMC stimulation in the presence		
		Culture medium (control)	M49-16	M49-16 delArcA	Culture medium (control)	M49-16	M49-16 delArcA
0 μM	Naïve	3.8 [3.1;4.2]	3.9 [3.4;4.1]	3.8 [3.3;4.5]	7.2 [6.0;10.9] ΔΔ	4.8 [4.3;7.3] ††	7.8 [6.4;12.0] §§
	CM	3.2 [2.6;3.9]	3.3 [2.7;4.2]	3.2 [2.5;4.8]	6.9 [5.6;11.2] ΔΔ	4.5 [3.5;8.4]	7.4 [6.1;11.7] §§
	EM	3.0 [2.6;3.5]	3.4 [2.5;3.9]	3.1 [2.6;4.4]	6.7 [5.1;8.3] ΔΔ	5.1 [3.7;7.3] †	7.2 [6.0;8.7] §
	TEMRA	3.4 [2.9;4.0]	3.9 [3.1;4.8]	3.5 [3.2;5.3]	5.8 [4.9;7.4] Δ	5.5 [4.3;7.8] †	6.3 [5.7;7.7] §
2 μM	Naïve	3.5 [2.9;4.1]	3.6 [3.2;4.4]	3.7 [3.2;4.2]	7.1 [6.0;11.5]	7.0 [6.0;10.3]	7.3 [6.1;11.1]
	CM	2.6 [2.2;3.9]	3.4 [2.5;5.4]	3.2 [2.5;4.3]	6.5 [5.8;12.0]	7.2 [6.5;12.1]	7.1 [5.7;11.4]
	EM	2.4 [2.1;3.8]	3.2 [2.4;5.6]	3.0 [2.3;4.2]	5.5 [4.9;9.6]	6.8 [5.5;11.6]	5.6 [4.6;9.4]
	TEMRA	2.9 [2.5;4.3]	4.0 [3.1;5.8]	4.0 [3.0;4.3]	5.2 [5.0;6.8]	6.1 [5.1;9.0]	4.7 [4.2;6.9]

Comments. Digitals 1, 2, 3 denote significance value at $p<0.05$, $p<0.01$, $p<0.001$, respectively.
Δ significant difference compared to control, without stimulation, in arginine-free settings;
† significant difference compared to M49-16, without stimulation, in arginine-free settings;
§ significant difference compared to M49-16delArcA, without stimulation, in arginine-free settings.

Table S12. Effects of parental and mutant strain-derived SDSC on CD4+ T cell autophagy (48-hour incubation).

**Arginine supplement**	**CD4+ Subsets**	**Intensity of autophagy (MFI, Med [Q25;Q75], n=13) after 48 hours PBMCs incubation in the presence**			**Intensity of autophagy (MFI, Med [Q25;Q75], n=13) after48 hours PBMC stimulation in the presence**		
		**Culture medium (control)**	**M49-16**	**M49-16 delArcA**	**Culture medium (control)**	**M49-16**	**M49-16 delArcA**
0 µM	Naïve	2.9 [2.3;3.4]	3.0 [2.6;3.2]	2.9 [2.7;3.4]	5.7 [4.2;6.3] ΔΔ	3.7 [3.5;4.8] ††	6.2 [4.3;7.4] §§
	CM	3.2 [2.8;3.7]	3.2 [3.0;3.8]	3.6 [3.3;4.1]	5.9 [4.8;7.0] ΔΔ	4.0 [3.6;4.8] † &	6.9 [4.4;8.4] § •
	EM	3.2 [2.7;4.2]	3.7 [3.3;3.9]	4.1 [3.6;4.3]	6.1 [5.9;9.6] ΔΔ	4.8 [3.6;5.2] &&	7.4 [5.5;8.5] §§ ••
	TEMRA	4.1 [3.3;4.9]	3.7 [3.6;4.2]	4.3 [3.8;4.9]	7.9 [6.7;9.5] Δ	4.9 [3.4;5.9] &	7.6 [6.3;8.2] §§ •
2 µM	Naïve	3.1 [2.5;3.4]	3.0 [2.9;3.4]	3.2 [2.4;3.4]	5.9 [4.3;7.8]	5.9 [4.5;6.9]	6.3 [4.3;7.7]
	CM	3.6 [2.9;3.9]	3.7 [3.5;4.4]	3.7 [3.2;3.9]	6.5 [4.9;8.3]	5.6 [5.5;7.3] ••	6.7 [4.7;8.4]
	EM	3.5 [3.1;4.0]	4.1 [3.8;5.5]	3.8 [3.5;4.1]	7.8 [6.8;8.7]	7.4 [6.8;10.4] ••	8.1 [6.1;8.8]
	TEMRA	4.0 [3.8;4.6]	4.2 [3.6;5.0]	4.0 [3.9;4.4]	8.0 [6.9;9.2]	8.2 $ [7.9;9.8] •	8.2 [7;9.9.0]

Comments. Digitals 1, 2, 3 denote significance value at $p<0.05$, $p<0.01$, $p<0.001$, respectively.
Δ significant difference compared to control, without stimulation, in arginine-free settings;
† significant difference compared to M49-16, without stimulation, in arginine-free settings;
§ significant difference compared to M49-16delArcA, without stimulation, in arginine-free settings;
& significant difference compared to control with stimulation in arginine-free settings;
• significant difference compared to M49-16, with stimulation, in arginine-free settings.

Table S13. Effects of parental and mutant strain-derived SDSC on CD4+ T cell autophagy (96-hour incubation).

Arginine supplement	CD4+ Subsets	Intensity of autophagy (MFI, Med [Q25;Q75], n=13) after 96 hour PBMCs incubation in the presence			Intensity of autophagy (MFI, Med [Q25;Q75], n=13) after 96-hour PBMC stimulation in the presence		
		Culture medium (control)	M49-16	M49-16 delArcA	Culture medium (control)	M49-16	M49-16 delArcA
0 μM	Naïve	3.0 [2.8;4.0]	3.2 [2.6;3.8]	3.0 [2.5;3.5]	5.6 [4.3;6.9] ΔΔ	3.9 [3.1;5.1]	5.3 [5.0;7.5] §§
	CM	3.3 [2.9;3.8]	3.4 [2.7;3.8]	3.1 [2.6;4.5]	6.3 [5.5;10.2] ΔΔ	3.7 [3.1;5.9]	7.2 [5.8;10.1] §§
	EM	2.8 [2.5;3.6]	3.1 [2.6;4.0]	2.9 [2.5;4.4]	6.7 [5.6;9.0] ΔΔ	4.2 [3.3;6.1]	7.4 [5.6;8.7] §§
	TEMRA	3.3 [3.0;4.1]	4.0 [3.5;4.7]	4.1 [2.7;4.8]	7.2 [5.8;8.1] Δ	5.6 [3.8;7.1]	6.7 [5.3;7.3] §
2 μM	Naïve	2.9 [2.5;3.3]	3.1 [2.4;3.6]	2.9 [2.3;3.4]	5.4 [4.3;8.1]	5.6 [4.3;7.9]	6.2 [3.9;7.4]
	CM	2.7 [2.3;3.7]	3.2 [2.3;5.0]	3.0 [2.5;3.9]	6.7 [5.5;10.9]	6.3 [5.5;10.6]	7.5 [5.6;10.4]
	EM	2.4 [2.1;3.8]	3.4 [2.6;6.0]	3.2 [2.4;4.1]	6.0 [4.8;9.5]	6.5 [5.3;10.5]	5.9 [5;8.9]
	TEMRA	3.2 [2.8;4.1]	4.2 [3.4;5.8]	4.1 [2.7;4.6]	6.2 [4.5;8.0]	6.4 [5.1;9.6]	5.6 [4.4;7]

Comments. Digitals 1, 2, 3 denote significance value at $p<0.05$, $p<0.01$, $p<0.001$, respectively.
Δ significant difference compared to control, without stimulation, in arginine-free settings;
§ significant difference compared to M49-16delArcA, without stimulation, in arginine-free settings.
